# Melatonin-micronutrients Osteopenia Treatment Study (MOTS): a translational study assessing melatonin, strontium (citrate), vitamin D3 and vitamin K2 (MK7) on bone density, bone marker turnover and health related quality of life in postmenopausal osteopenic women following a one-year double-blind RCT and on osteoblast-osteoclast co-cultures

**DOI:** 10.18632/aging.101158

**Published:** 2017-01-26

**Authors:** Sifat Maria, Mark H. Swanson, Larry T. Enderby, Frank D'Amico, Brianna Enderby, Rebekah M. Samsonraj, Amel Dudakovic, Andre J. van Wijnen, Paula A. Witt-Enderby

**Affiliations:** ^1^ Division of Pharmaceutical Sciences, Duquesne University School of Pharmacy, Pittsburgh, PA 15282, USA; ^2^ Functional Medicine, Heart Preventics, LLC, Sequim, WA 98382, USA; ^3^ Enderby Healthcare/Legal Consulting, LLC, Pittsburgh, PA 15102, USA; ^4^ Department of Mathematics, Duquesne University School of Liberal Arts, Pittsburgh, PA 15282, USA; ^5^ Mayo Clinic, Department of Orthopedic Surgery, Rochester, MN 55905, USA

**Keywords:** osteopenia, melatonin, osteoblasts, osteoclasts, PPARγ, GLUT4, adipocytes

## Abstract

This one-year double blind randomized control trial assessed the effects of nightly melatonin, strontium (citrate), vitamin D3 and vitamin K2 (MK7; MSDK) on bone mineral density (BMD) and quality of life (QOL) in postmenopausal osteopenic women (ages 49-75). Compared to placebo, MSDK treatment increased BMD in lumbar spine (4.3%) and left femoral neck (2.2%), with an upward trend for total left hip (p=0.069). MSDK increased serum P1NP levels and reduced bone turnover (CTx:P1NP). Psychometric analyses indicated that mood and sleep quality improved for the MSDK group. MSDK-exposed human mesenchymal stem cells (hMSCs) and human peripheral blood monocytes (hPBMCs) plated in transwells or layered demonstrated increases in osteoblastogenesis, decreases in osteoclastogenesis, increases in OPG (TNFRSF11B) and decreases in RANKL (TNFSF11) levels. In transwell osteoblasts, MSDK increased pERK1/2 (MAPK1/MAPK3) and RUNX2 levels; decreased ERK5 (MAPK7); and did not affect the expression of NFκB (NFKB1) and β1integrin (ITGB1). In layered osteoblasts, MSDK also decreased expression of the metabolic proteins PPARγ (PPARG) and GLUT4 (SLC2A4). In adipose-derived human MSCs, MSDK induced osteoblastogenesis. These findings provide both clinical and mechanistic support for the use of MSDK for the prevention or treatment of osteopenia, osteoporosis or other bone-related diseases.

## INTRODUCTION

The menopausal transition, although a natural physiological phenomenon, is often accompanied by bone loss leading to osteoporosis and related fractures. Currently, about 9 million US adults suffer from osteoporosis and another 50 million from osteopenia. These numbers are predicted to increase up to 11.9 million for osteoporosis and 64.3 million for osteopenia by 2030 [1]. One in three women and one in five men over age 50 will experience an osteoporosis-related fracture worldwide [2]. Over half of all women in the U.S. above age 50 have osteopenia with a prevalence of approximately 3.4 times more than osteoporosis [3]. Consequently, twice the number of fractures arise from women with osteopenia as they represent almost 50% of the total population at risk [4]. Besides bone loss, women transitioning through menopause also experience disrupted sleep, depressed mood, higher levels of anxiety and stressful life events [5].

Osteopenia is defined as below normal bone density and the precursor to osteoporosis, with a T-score −1 to −2.5 [1]. During the osteopenic period, bone loss usually progresses insidiously and unnoticed unless a fracture occurs. If a Dual Energy X-ray Absorptiometry (DXA) is performed, it can serve as the baseline bone mineral density (BMD) assessment for diagnosis and future monitoring. However, pharmacological treatment is typically delayed or given “watchful waiting” until the transition to osteoporosis is diagnosed (T-score −2.5 or less at the femoral neck/spine); or if there is a history of a previous hip or vertebral fracture; or if the T score is between −1.0 and −2.5 at the femoral neck/spine and the 10-year risk of hip fracture ≥ 3% or 10-year risk of major osteoporosis-related fracture ≥ 20% by Fracture Risk Assessment Tool (FRAX^®^) calculation. Based on these current guidelines, a majority of postmenopausal osteopenic women may not receive their first DXA screening until age 65 [6]. Thus, osteopenia often carries a significant treatment uncertainty during the time of the greatest fracture risk burden [3].

The non-pharmacologic recommendation for osteopenia include calcium or vitamin D supplementation, which has proven to be largely ineffective for reducing the incidence of fractures or the prevention of osteoporosis. Also the combination of calcium plus vitamin D only slightly reduces the risk of hip and other fractures [7, 8]. Most of the existing pharmaceutical bone loss therapies are treatment-focused rather than preventative and mainly target preventing further bone loss rather than enhancing new bone formation. A new non-invasive treatment focusing on bone anabolism during osteopenia would be a critical first step to begin as early as perimenopause for maintaining normal bone integrity and microarchitecture and to prevent future fractures [9]. In this study, we investigated whether our treatment- melatonin, strontium (citrate), vitamin D_3_ and vitamin K_2_ (MSDK) could provide a much-needed stopgap treatment and serve as a safe and effective modulator of anabolic bone formation and metabolism to improve the efficacy of pre-osteoporosis prevention. In the Melatonin-micronutrients Osteopenia Treatment Study (MOTS; NCT01870115), we examined whether melatonin added in combination with three other natural bone-protective micronutrients: strontium (citrate), vitamins D_3_ and K_2_ (MSDK) could improve bone health without affecting or even improving health related quality of life (hrQOL) in postmenopausal osteopenic women. The second goal was to identify potential mechanisms underlying MSDK's action using human osteoblast and osteoclast co-culture models. Women with osteopenia were targeted because osteopenia accounts for the highest population burden of fractures [4, 10]. A nutritional bone support supplement formulated with MSDK could address this population group and become readily available without prescription at a nominal cost.

Melatonin is a chronotherapeutic hormone and nutritional supplement that has demonstrated efficacy to renormalize bone marker turnover in perimenopausal women [11] and increase bone density in post-menopausal women with osteopenia [12]. Strontium increased vertebral and femoral bone density and reduced fracture in both postmenopausal osteopenic and osteoporotic women [13, 14]. Vitamin K_2_ has been shown to prevent bone loss, reduce the incidence of vertebral fractures and modestly increase bone density in a postmenopausal cohort primarily by improving bone microarchitecture and the bone mineral/matrix ratio and also by preserving bone quality [15, 16]. Combination of vitamin D_3_ with K_2_ has been shown to increase vertebral bone density in postmenopausal women [17, 18]. Also, improved vitamin D_3_ status in postmeno-pausal women has been shown to significantly enhance BMD response to strontium ranelate, particularly in the femoral neck [19]. The Combination of Micronutrients for Bone (COMB) study, using strontium (citrate) and vitamins D_3_ and K_2_ was at least as effective as bisphosphonates and strontium ranelate at increasing BMD in the hip, spine and femoral neck sites [20].

The MOTS utilizes a safe, complementary, non-pharmacologic combination therapy based on the hypothesis of *chronosynergy* - novel treatment approach using several condition-targeted bone restorative agents with melatonin to reverse bone loss reducing the need for osteoporosis-related medications later. In addition to improving bone health outcomes, this novel therapy may reduce the cost of treating bone disease. In a recent study, the introduction of melatonin into an osteoporosis treatment formulary resulted in significant savings with respect to annual treatment costs [21] lowering the economic burden associated with bone loss therapies. With continued study and validation, MSDK could become an early treatment option in the time-course for managing postmenopausal and age-related bone loss.

## RESULTS

### Recruitment

**Figure 1:**
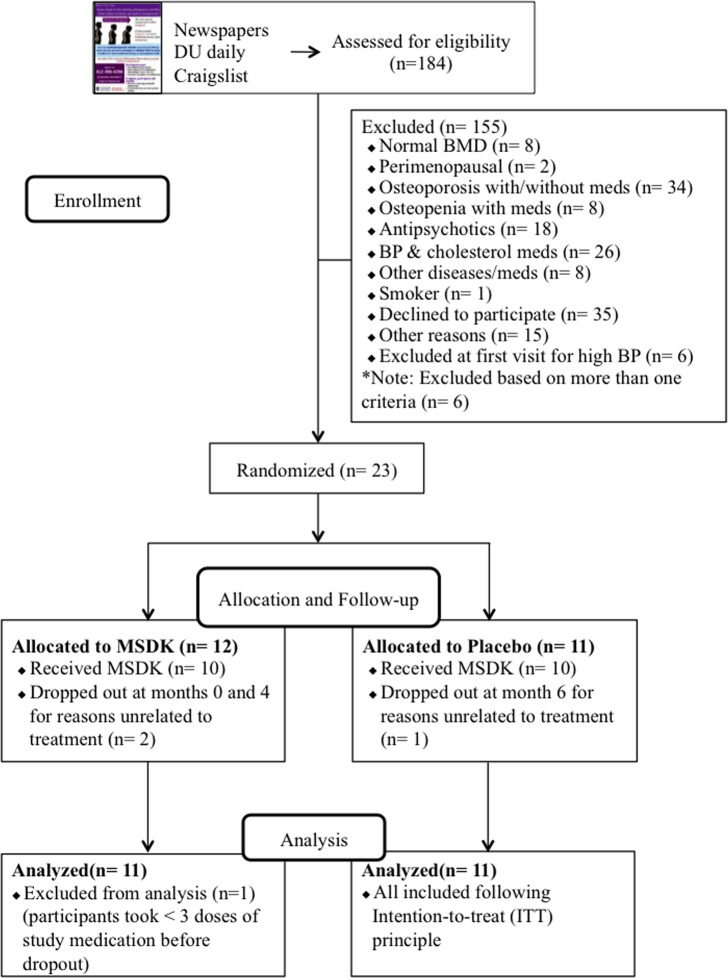
Flow chart of study subject recruitment and enrollment

Figure 1 illustrates the summary of the participants' screening and enrollment processes. A total of 184 women responded of which 105 (57.1%) did not meet the inclusion and exclusion criteria. Individuals were excluded due to having either normal bone density T-score (7.6%), or being perimenopausal (2%), or by having osteoporosis with or without taking medications (32.4%), or being osteopenic but taking bisphospho-nates or hormone therapy (7.6%). Other common reasons for exclusion included use of medications for blood pressure (25%), depression and anxiety (17%); having diseases such as ulcerative colitis, rheumatoid arthritis (7.6%); or smoking (1%). Among the 79 respondents invited to join the study, 29 (37%) came for the initial visit and the other 50 individuals (63%) chose not to participate for their own reasons such as the fear of being on placebo for one year (35%) or not being able to commit to the bimonthly visits (25%). At the initial appointment, another 6 women were excluded due to undiagnosed hypertension. Finally, 23 women were enrolled in the study and randomized into either placebo or treatment (MSDK). One subject withdrew the next day and was not included in the overall analysis because it had been determined that if a subject took <3 doses of the study supplements, then they would be replaced and not included in the analysis. Another two subjects (one from MSDK and one from placebo) withdrew at months 4 and 6, respectively. They were included in the analysis following intention-to-treat (ITT) principle. All women were self-identified as white.

Our recruitment strategies resulted in a healthy postmenopausal population with an age range [58.6 yr. (49-75)] and body composition relevant for their age and physical status. Their DXA and FRAX^®^ scores represented an osteopenic population, with moderate risk of fracture. Except for baseline serum collagen type I cross- linked telopeptide (CTx) values and diastolic blood pressure in the MSDK cohort, all other parameters were similar between groups suggesting an overall and well-adjusted randomization. Psychological assessment suggested that all subjects had normal mental health with no significant anxiety, stress or depression. Nearly 87% subjects were taking either calcium/vitamin D_3_, multivitamins and/or other dietary supplements. Almost all subjects were healthy sleepers with active lifestyles (Table 1).

**Table 1:** Baseline characteristics of study cohort un-stratified and stratified by treatment

	Un-stratified	Stratified	
		Placebo (n=11)	MSDK (n=11)
Age	58.6 ± 1.12 (49-75)	57 ± 1.41 (49-63)	60 ± 1.73 (54-75)
Blood pressure (BP)			
Systolic (mmHg)	118.1 ± 2.47 (100-140)	118.8 ± 3.29 (100-140)	117.4 ± 3.83 (100-140)
Diastolic (mmHg)	73.4 ± 1.24 (61-80)	70.91 ± 1.76 (61-80)	75.91 ± 1.44 (64-80)*
Height (cm)	166.6 ± 1.64 (154.9-182.9)	170.2 ± 2.13 (158.8-182.9)	163 ± 2.06 (154.9-172.7)
Weight (lb)	144.6 ± 4.83 (113.4-198)	147.9 ± 6.68 (121.4-177.2)	141.2 ± 7.16 (113.4-198)
BMI (kg/m^2^)	23.62 ± 0.7 (19.5-31.7)	23.16 ± 0.93 (19.6-28)	24.09 ± 1.08 (19.5-31.5)
BMR (kcal/24h)	1319 ± 23 (1162-1543)	1347 ± 31.06 (1215-1509)	1291 ± 33.16 (1162-1543)
Fat Mass (lb)	47.84 ± 3.51 (24.2-91.6)	48.96 ± 4.39 (33.2-75.6)	46.71 ± 5.67 (24.2-91.6)
Fat (%)	32.3 ± 1.32 (21.4-46.3)	32.52 ± 1.59 (25.3-43.2)	32.08 ± 2.18 (21.4-46.3)
Lean Body Mass (lb)	96.75 ± 1.81 (83-112.4)	98.98 ± 2.99 (83-112.4)	94.51 ± 1.94 (85.8-106.4)
Total Body Water (lb)	70.8 ± 1.32 (60.8-82.2)	72.45 ± 2.18 (60.8-82.2)	69.14 ± 1.42 (62.8-77.8)
BMD (DXA)			
Femoral Neck (g/cm^2^)	0.641 ± 0.01 (0.511-0.717)	0.641 ± 0.02 (0.511-0.717)	0.636 ± 0.01 (0.598-0.701)
Total Left Hip (g/cm^2^)	0.768 ± 0.01 (0.641-0.859)	0.769 ± 0.02 (0.641-0.859)	0.761 ± 0.01 (0.704-0.845)
Lumbar Spine (g/cm^2^)	0.865 ± 0.02 (0.771-1.108)	0.869 ± 0.03 (0.782-1.108)	0.852 ± 0.02 (0.771-0.938)
Achilles heel T-score	−1.2 ± 0.21 [(−2.7)-(1.8)]	−1.14 ± 0.36 [(−2.2)-(1.8)]	−1.27 ± 0.24 [(−2.7)-(0.1)]
Previous fractures	8 (36%)	4 (36%)	4 (36%)
FRAX: major Osteoporotic	12.02 ± 0.96 (6.1-24)	13.05 ± 1.59 (6.1–24)	10.98 ± 1.07 (6.7–16)
FRAX: Hip	1.71 ± 0.33 (0.2-7.4)	1.92 ± 0.61 (0.5–7.4)	1.51 ± 0.28 (0.2–3.2)
Bone markers			
P1NP (pg/mL)	45.71 ± 5.24 (4.75-100.5)	46.41 ± 6.31 (15.23-83.87)	44.95 ± 8.91 (4.75–100.5)
Osteocalcin (ng/mL)	24.68 ± 1.95 (9.78-52.89)	24.55 ± 2.51 (9.77–36.03)	24.82 ± 3.11 (16.17–52.9)
CTx (ng/mL)	8.78 ± 0.85 (3.89-18.97)	7.08 ± 0.84 (3.89-12.31)	10.48 ± 1.33 (4.6–18.97)*
Vitamin D_3_ (ng/mL)	16.72 ± 4.71 (0.5-100.9)	20.77 ± 8.77 (0.83–100.9)	12.68 ± 3.59 (0.5–38.61)
CRP (ng/mL)	2334 ± 962.7 (124.1-17974)	2018 ± 1132 (124.1–11705)	2621 ± 1573 (124.1–17974)
MENQOL			
Vasomotor	2.71 ± 0.36 (1-6)	2.61 ± 0.56 (1–6)	2.82 ± 0.47 (1–5)
Physical	2.72 ± 0.27 (1.05-5.55)	2.75 ± 0.36 (1.1–5.5)	2.69 ± 0.42 (1.05–5.3)
Psychosocial	2.61 ± 0.31 (1-6.67)	2.8 ± 0.53 (1–6.67)	2.42 ± 0.36 (1–4.5)
Sexual	2.94 ± 0.39 (1-6.67)	2.97 ± 0.49 (1-5)	2.91 ± 0.63 (1–6.67)
STAI: State	30.82 ± 2.28 (20-58)	28.45 ± 3.34 (20–58)	33.18 ± 3.11 (20–53)
STAI: Trait	34.41 ± 2.25 (20-65)	33.82 ± 3.71 (22–65)	35 ± 2.75 (20–45)
PSS	11.73 ± 1.71 (1-30)	11.45 ± 2.39 (1–30)	12 ± 2.56 (2–26)
CES-D	6.41 ± 1.63 (0-37)	7.09 ± 3.09 (0–37)	5.73 ± 1.21 (1–13)
Calcium vitaminD_3_/Multivitamins	19 (86.4%)	10 (90%)	9 (81.8%)

Mean ± S.E.M. (Range). BMI= Body mass index; BMR= Basal metabolic rate; BMD= Bone mineral density; P1NP= Total procollagen type 1 amino-terminal propeptide; OC= Osteocalcin; CTx= Collagen Type I C-Telopeptide; MENQOL= Menopause specific Quality Of Life; STAI= State and Trait Anxiety Inventory; PSS= Perceived Stress Scale; CES-D= Center for Epidemiologic studies Depression.

* *p* < 0.05 versus placebo.

### MSDK increases bone mineral density (BMD) in postmenopausal osteopenic women

**Figure 2:**
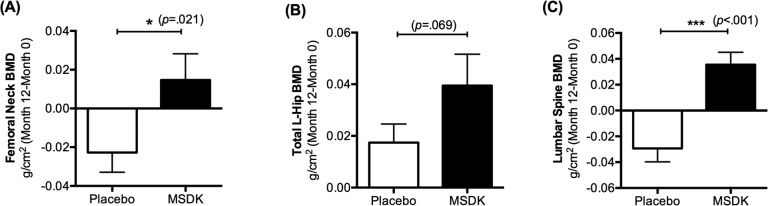
Treatment effects on bone mineral density (BMD) in placebo and MSDK groups Bone mineral density in the left femoral neck, total left hip and lumbar spine was measured via DXA (n=11 per group). Each bar represents the mean (± S.E.M.) change in bone mineral density (g/cm^2^) from baseline to month 12 in the (**A**) left femoral neck, (**B**) total left hip and (**C**) lumbar spine (L1-L4) area, respectively for placebo (open bar) and MSDK (closed bars). *p ≤ 0.05 and ***p ≤ 0.001 versus placebo, unpaired one tailed *t*-test with Welch's correction.

Bone health was monitored over the course of the study by DXA; by assessing serum bone marker activity; and by measuring fracture risk probability using FRAX^®^. Figure 2 indicates the mean change in BMD (g/cm^2^) from baseline to month 12 in the left femoral neck, total left hip and lumbar spine. In left femoral neck, participants in the MSDK group had an average BMD change of +0.015 (2.2%) over one year, whereas participants in the placebo group had an average BMD change of −0.023 (−3.6%). Data analyses showed significant differences between groups (*p=* 0.021) (Fig. 2A). In total left hip, MSDK and placebo group had average BMD changes of +0.039 (5.03%) and +0.017 (2.2%), respectively. Even though the analysis showed no significant difference in BMD between groups in this area, a trend (*p*= 0.069) for improvement in the MSDK group existed (Fig. 2B). Women taking MSDK showed the most significant increase in BMD in the lumbar spine (L1-L4) area (*p*< 0.001 vs. placebo). Mean BMD changes in this area for MSDK and placebo groups were +0.035 (4.3%) and −0.029 (−3.2%), respectively (Fig. 2C). Corresponding T-score changes in the left femoral neck, total left hip and lumbar spine area are shown in Table 2. FRAX^®^ assessments revealed that one year MSDK treatment significantly reduced major vertebral and non-vertebral osteoporotic fracture risk by 6.48% from baseline compared to the 10.82% increase observed in placebo (*p=* 0.008). No significant effects of MSDK on hip fracture risk were observed.

**Table 2:** Treatment effects on bone density (T-scores), fracture risk probability (FRAX), body composition, psychometric parameters and general well-being

	Placebo				MSDK		
	**Month 0**	**Month 12**	**Change**	**Month 0**	**Month 12**	**Change**
T-Scores						
Femoral Neck	−1.87	−2.13	**−0.20 (0.09)**	−1.88	−1.74	**0.13 (0.12)** *
Total Left Hip	−1.42	−1.33	0.16 (0.06)	−1.37	−1.02	0.35 (0.09)
Lumber Spine L1-L4	−1.61	−1.81	**−0.25 (0.09)**	−1.70	−1.39	**0.31 (0.09)** ***
Achilles heel BMD (T-score)	−1.14	−1.29	−0.18 (0.13)	−1.27	−1.36	0.09 (0.11)
FRAX						
Major Osteoporotic	13.05	14.79	**1.73 (0.44)**	10.98	10.6	**−0.38 (0.56)** **
Hip	1.92	2.84	0.92 (0.33)	1.51	1.45	0.11 (0.31)
Height (cm)	170.18	169.82	−0.35 (0.18)	163.02	162.95	−0.08 (0.25)
Weight (lb)	147.95	148.71	0.76 (1.71)	141.22	143.31	1.20 (0.83)
BMI (kg/m^2^)	23.16	23.36	0.21 (0.32)	24.09	24.31	0.24 (0.16)
BMR (kcal/24h)	1347.36	1347.82	0.45 (7.37)	1291.27	1293.91	2.64 (3.32)
Fat Mass (lb)	48.96	49.8	0.84 (1.88)	46.71	47.78	1.07 (0.74)
Fat (%)	32.52	32.85	0.33 (0.89)	32.08	32.59	0.51 (0.54)
Lean Body Mass (lb)	98.98	98.9	−0.07 (0.63)	94.51	94.53	0.02 (0.97)
Total Body Water (lb)	72.45	72.38	−0.07 (0.46)	69.14	69.22	0.07 (0.73)
MENQOL						
Vasomotor	2.61	2.30	−0.31 (0.48)	3.00	2.53	−0.47 (0.28)
Physical	2.75	2.43	−0.32 (0.24)	2.53	2.18	−0.34 (0.34)
Psychosocial	2.80	2.27	−0.53 (0.30)	2.22	2.10	−0.12 (0.39)
Sexual	2.97	2.70	−0.27 (0.31)	2.93	2.03	−0.90 (0.49)
STAI: Anxiety						
State	28.45	28.4	−0.05 (1.09)	32.5	27.4	−5.10 (4.08)
Trait	33.82	30.1	−3.71 (1.80)	34.1	31.3	−2.80 (3.38)
PSS: Perceived stress	11.45	7.6	−3.85 (1.18)	11.5	10.7	−0.80 (2.03)
CES-D: Depression	7.09	5.2	−1.89 (0.86)	5.1	5	−0.10 (1.67)
Medication intake	90.03%			92.4%		
Sleep duration (hr)	7.06 ± 0.23 (range: 6.13-8.31)			6.85 ± 0.27 (range: 4.85-7.97)		
Exercise intensity	435 ± 77.93 (range: 209-1036)			300.2 ± 75.78 (range: 161-1044)		
Multivitamins/OTC supplements	81.82%			81.82%		

Mean (± S.E.M.). BMD= Bone mineral density; BMI= Body mass index; BMR= Basal metabolic rate; MENQOL= Menopause specific Quality Of Life; STAI= State and Trait Anxiety Inventory; PSS= Perceived Stress Scale; CES-D= Center for Epidemiologic Studies Depression.

* *p* < 0.05

** *p* < 0.01

*** *p* < 0.001 versus placebo (n=11 per group).

### MSDK increases serum bone formation markers in postmenopausal osteopenic women

**Figure 3:**
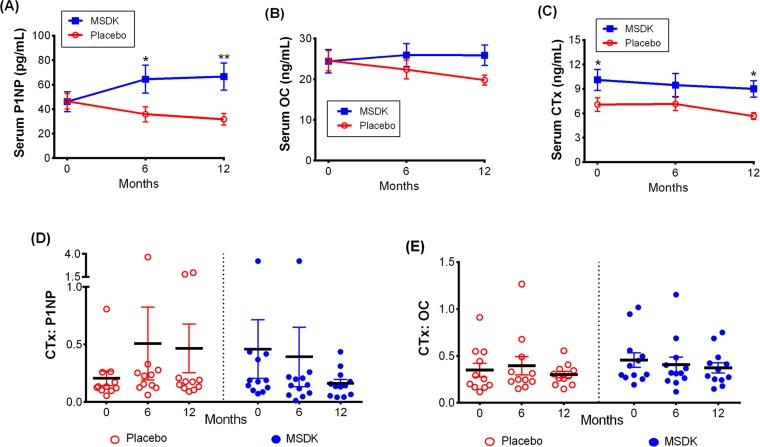
Treatment effects on serum bone marker turnover in placebo and MSDK groups Bone formation markers (**A**) total procollagen type 1 amino-terminal propeptide (P1NP) and (**B**) osteocalcin (OC; both intact and N-terminal mid-fragments), serum bone resorption marker (**C**) Collagen Type I C-Telopeptide (CTx) were measured at months 0, 6 and 12. Serum samples were collected from participants (n=11 per group) and bone markers concentrations were measured by sandwich ELISA assay using commercially available specific bone marker kits at all three time points. Each point in the line graph represents the least square mean (± S.E.M.) in the bone markers concentration for placebo (open circle, red) and treatment (closed box, blue). Scatter plots represent the ratio of (**D**) CTx: P1NP and (**E**) CTx: OC for each study subject, respectively, where the solid lines indicate the mean (± S.E.M.) value for each group. *p ≤ 0.05 and **p ≤0.01 versus placebo at similar time points. Longitudinal analysis for repeated measures using a generalized linear mixed model (GLMM) approach, considering groups and times as fixed effects and subjects nested within the groups as random.

Participants' serum bone formation markers total procollagen type 1 amino-terminal propeptide (P1NP) and osteocalcin (OC; both intact and N-terminal mid-fragments) and the bone resorption marker collagen type I cross- linked telopeptide (CTx) were assessed every 6 months throughout the study to evaluate the change in bone marker status with treatment (Fig. 3). MSDK treatment significantly increased serum P1NP vs. placebo at month 6 (*p*= 0.023) and month 12 (*p*= 0.004). Serum P1NP levels varied widely within and between groups ranging from 25.04 to 151.9 pg/mL (average: 66.62 ± 11.09) in MSDK and from 2.75 to 48.14 pg/mL (average: 31.76 ± 4.75) in placebo at month 12 (Fig. 3A). Further analysis revealed that the mean increase in P1NP levels in the MSDK group primarily occurred during the first six months of the study (data not shown). Even though serum OC levels did not differ significantly between groups at any time, a gradual decrease in the level was observed in placebo but not in the MSDK group (*p*= 0.071 vs. MSDK at month 12). MSDK and placebo groups had average OC levels of 25.88 (± 2.5) ng/mL and 19.79 (± 1.21) ng/mL, respectively at month 12 (Fig. 3B). Even though serum CTx levels were significantly higher in the MSDK group at baseline, it remained steady throughout the study suggesting that either MSDK had no intrinsic effect on CTx or the dose of MSDK was not high enough to compensate for the higher baseline CTx level in this group. Average CTx levels at month 12 in MSDK and placebo groups were 8.99 (± 1.01) ng/mL and 5.65 (± 0.42) ng/mL, respectively (Fig. 3C).

Because osteoclast activity is tightly coupled to osteoblast activity [22], bone turnover was assessed by measuring the ratio of bone resorption (CTx) to bone formation (P1NP or OC). Despite having higher baseline CTx levels in the MSDK group compared to placebo, the ratio of CTx:P1NP in women taking MSDK demonstrated a time-dependent decrease whereas no time-dependent increases were observed in placebo (Fig. 3D). Though not significant, a similar trend was observed for CTx:OC (Fig. 3E). In summary, one year MSDK treatment significantly increased BMD in the left femoral neck and lumbar spine, with a trend towards improvement in the total left hip. Increased BMD was associated with increases in P1NP, decreases in bone marker turnover (↓CTx:P1NP) and a reduction in the 10-year probability of major osteoporotic fracture risk.

### MSDK increases melatonin-sulfate levels in postmenopausal osteopenic women

**Figure 4:**
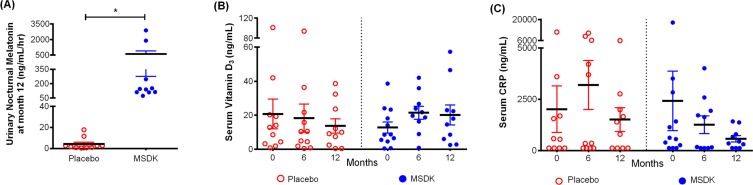
Treatment effects on urinary nocturnal melatonin, serum vitamin D_3_ and serum C-reactive protein (CRP) level in placebo and MSDK groups (**A**) Nocturnal hourly melatonin secretion in the urine was measured at month 12 (n=10 per group), by collecting all urine samples between 10pm and 6am and and measuring using urinary melatonin-sulfate ELISA kit. Each point in the scatter plots represents an individual's urine melatonin-sulfate level, in placebo (open circle, red) or MSDK (closed circle, blue). *p ≤ 0.05 versus placebo at month 12; Unpaired one-tailed *t*-test with Welch's correction. Serum levels of (**B**) Vitamin D_3_ and (**C**) CRP were measured at months 0, 6 and 12, using specific ELISA kits. Each point in the scatter plots represents the concentration for a single subject at a specific time point, in the placebo (open circle, red) or MSDK (closed circle, blue). *p ≤ 0.05 versus placebo at similar time point; Longitudinal analysis for repeated measures using generalized linear mixed model (GLMM) approach, considering groups and times as fixed effects and subjects nested within the groups as random.

Figure 4 illustrates nocturnal urinary melatonin-sulfate levels assessed at month 12 as well as serum vitamin D_3_ and C-reactive protein (CRP) levels assessed at months 0, 6 and 12. As shown in Figure 4A, women taking MSDK had significantly higher levels of urinary melatonin-sulfate levels compared to placebo (*p*= 0.0463). In placebo, urinary melatonin-sulfate levels ranged from 0.43 to 17.69 ng/mL/hr (average: 4.19 ± 1.83). Participants in the MSDK group showed wide variation in their nighttime melatonin-sulfate levels ranging from 73.13 to 2883 ng/mL/hr (average: 586.4 ± 309.4) despite taking equal nightly doses. Melatonin levels were found to significantly correlate with lumbar spine BMD (*p*= 0.029; correlation co-efficient, r= 0.487; 95% CI= 0.0566 to 0.7648) supporting the requirement of daily melatonin for maintaining bone density.

Participants' serum vitamin D_3_ levels were assessed at months 0, 6 and 12 (Fig. 4B). Even though participants were allowed to take up to 1000IU of a vitamin D3 supplement for ethical reasons regardless of treatment, no significant differences were observed between group at baseline and over each timepoint. However, both groups showed wide variability in their serum vitamin D_3_ levels throughout the study ranging from 2.54 to 57.32 ng/mL (average: 20.19 ± 5.84) in MSDK and from 0.5 to 38.59 ng/mL (average: 13.71 ± 4.24) in placebo at month 12. Correlations between vitamin D_3_ levels and bone resorption were performed since it was shown that low vitamin D_3_ is associated with high CTx [23]. Similar to past-published studies, serum D_3_ levels in our study negatively correlated with CTx levels (*p*= 0.024; r = −0.5011; 95% CI= −0.7724 to −0.0751). As shown in Figure 4C, serum CRP levels varied widely within and between groups ranging from 124 to 1422 ng/mL (average: 573.1 ± 154.7) in MSDK and from 124.1 to 6023 ng/mL (average: 1513 ± 581.2) in placebo at month 12. Although serum CRP levels did not differ significantly within or between groups, those taking MSDK had low serum CRP at month 12, indicating a favorable effect of MSDK on inflammatory status.

### MSDK improves morphometric and psychometric parameters in postmenopausal osteopenic women

Morphometric analysis revealed that MSDK had no effect on overall body composition of subjects (Table 2); however, average height losses in MSDK and placebo groups were 0.08 cm and 0.35 cm, respectively (*p*= 0.38). Participants in the MSDK group showed less fluctuation in their weight change (F=4.248, DFn=10; *p*= 0.032), BMI change (F=4.112, DFn=10; *p*= 0.036), BMR change (F=4.936, DFn=10; *p*= 0.019) and fat mass change (F=6.409, DFn=10; *p*= 0.007) from baseline to month 12 compared to placebo. The lack of weight variation over time may produce favorable effects on bone mass as described [24].

**Figure 5:**
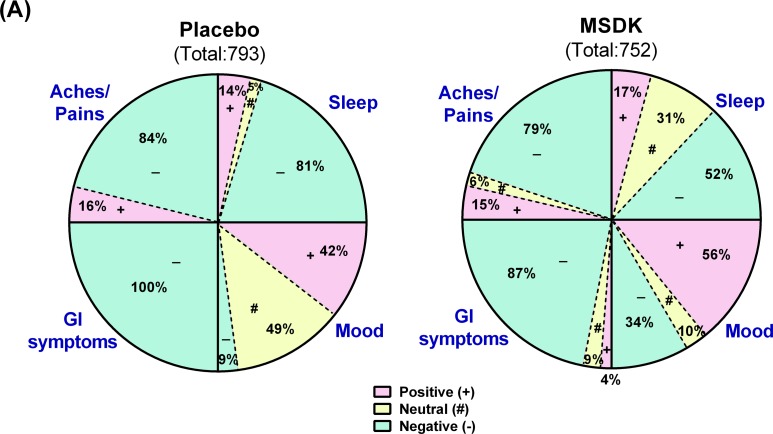
Treatment effects on participants' sleep quality, mood, GI upset and general aches/pains in placebo and MSDK groups (**A**) Total diary comments made by the participants in each group throughout the study were stratified into four categories: sleep, mood, GI upset and general aches/pains, as illustrated by the four segments in the pie diagram. Each category was sub-stratified as positive (pink), neutral (yellow) and negative (green) comments. Each portion represents the percent of total comments made under each category.

Psychometric analyses were conducted using validated questionnaires. No effect of MSDK on menopause quality of life, anxiety, stress or depression status were observed compared to placebo (Table 2). However, sexual domain scores began to move in opposite directions where a trend towards an improvement was observed in women taking MSDK and a trend towards worsening was observed in placebo (graph not shown). Sleep quality improved in women taking MSDK (Fig. 5A) indicated by 29% more positive/neutral comments made in the MSDK group vs. placebo (*p*< .0001). The relative risk ratio of a positive sleep statement for women taking MSDK was 2.5 (95% CI= 1.44 to 4.35), suggesting that the likelihood of a positive sleep statement is 150% higher in MSDK group compared to placebo. Although the women in MSDK group experienced an improvement in sleep quality, their sleep duration remained similar to placebo (Table 2). Average sleep time (in hours) for those taking MSDK or placebo was 6.85 hr and 7.06 hr, respectively. Regarding mood, more positive comments were made in the MSDK group (56%) compared to placebo (42%). Interestingly, all (100%) comments made about GI symptoms in the placebo group were negative whereas 87% were negative in the MSDK group—a reduction of 13%. Regarding general aches and pains, the percentage of negative comments made in the MSDK group was 79% compared to 84% recorded for placebo. Statistical analyses revealed no significant impact of MSDK on GI symptoms and general aches/pains. Most of what was reported, positive or negative, had to do with their overall general well-being reported in their daily diaries (Fig. 5A).

Analysis of exercise time and intensity showed no significant differences between groups (*p*= 0.23). Most participants in the placebo group were involved in high intensity exercise whereas almost all MSDK participants were involved in light to moderate exercise. Both groups also had an equal intake of multivitamins/herbal supplements/OTC products (81.82%). Analysis of confounders revealed no significant differences between groups (Table 2).

The fact that MSDK treatment did not worsen any of the subjective measures assessed could impact positively on compliance, which was high in our study (Placebo=90.03% and MSDK = 92.4%; Table 2). No incidence of adverse effects was reported from our participants in either group during their bimonthly check-ups or through their daily logs. Bimonthly assessment of blood pressure demonstrated that both systolic and diastolic levels remained in the normal range throughout the study even though baseline diastolic BP in the MSDK group was significantly higher than placebo (data not shown).

### MSDK increases osteoblastogenesis and decrease osteoclastogenesis in co-cultures of hMSCs and hPBMCs

**Figure 6:**
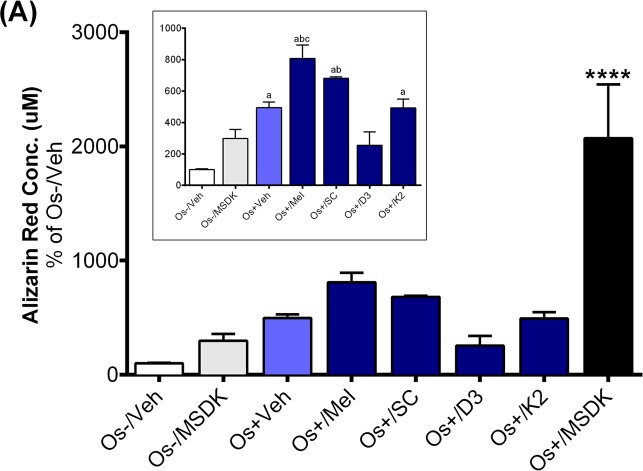

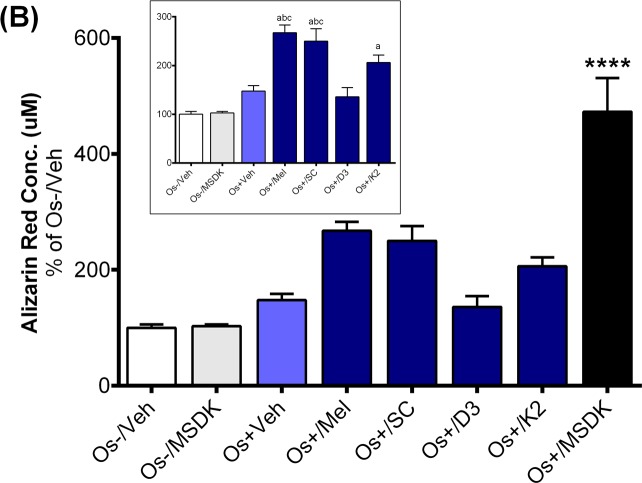
Effect of MSDK on osteoblast-mediated calcium mineralization On day 21 of MSDK exposure, calcium deposition by differentiated, matured osteoblasts were evaluated via alizarin red staining on the (**A**) bottom chamber cells of transwell co-culture and (**B**) layered co-culture. Inset graph represents similar analysis in absence of Os+/MSDK. Each bar represents the mean concentration of alizarin red (μM) for respective groups normalized against Os-/Veh (n=3; Transwell co-culture: ****=*p*<.0001 vs. all groups, a=*p*<.01 vs. Os-/Veh, b=*p*<.05 vs. Os-/MSDK, c=*p*<.05 vs. Os+/Veh; Layered co-culture: ****=*p*<.0001 vs. all groups, a=*p*<.01 vs. Os-/Veh, b=*p*<.01 vs. Os-/MSDK, c=*p*<.01 vs. Os-/Veh ). Os- =basal media, Os+ =osteogenic media, Veh= vehicle, Mel= melatonin, SC= strontium citrate, D3= vitamin D_3_ (Cholecalciferol), K2= vitamin K_2_ (MK7).

The increases in serum P1NP in the MSDK group could be the result of MSDK-mediated increases in osteoblastogenesis and decreases in osteoclastogenesis as demonstrated in the co-culture studies. Both alizarin red staining and tartrate resistant acid phosphatase

(TRAP) assays indicated successful differentiation of human adult mesenchymal stem cells (hMSCs) into osteoblasts and human peripheral blood monocytes (hPBMCs) into osteoclasts when grown together as co-culture in presence of osteogenic (Os+) media. Figure 6A represents calcium mineralization of osteoblasts grown in transwell co-culture. Human adult MSCs grown in growth media alone (Os-/Veh) or in presence of MSDK (Os-/MSDK) did not differentiate into osteoblasts as revealed by the absence of alizarin red staining. However, hMSCs exposed to osteogenic media containing MSDK (Os+/MSDK) differentiated into osteoblasts. We next investigated if the individual components of MSDK were capable of inducing osteoblast differentiation beyond that of osteogenic medium (Os+) alone. Melatonin, strontium citrate (SC), vitamin D_3_ (D3) or vitamin K_2_ (K2) were added to osteogenic (Os+) medium and alizarin red staining was measured. Melatonin was the only component that increased transwell osteoblast differentiation vs. osteogenic medium containing vehicle (Os+/Veh) (Fig. 6A inset). Similar treatment effects on osteoblast differentiation were observed in the layered co-culture except that the extent of MSDK-mediated osteoblast differentiation was less compared to the transwell osteoblasts (Fig. 6B) and both melatonin and strontium (citrate) induced osteoblast differentiation alone when compared to osteogenic medium containing vehicle (Os+/Veh) (Fig. 6B inset).

**Figure 7:**
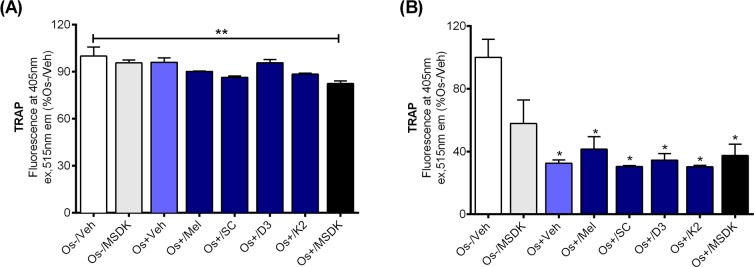
Effect of MSDK on osteoclast differentiation On day 21 of MSDK exposure, TRAP releasing activity by differentiated, mature osteoclasts was evaluated by quantitative Tartrate Resistant Acid Phosphatase (TRAP) assay on the (**A**) top chamber cells of transwell co-culture and (**B**) layered co-culture. Each bar represents the mean fluorescence reading of TRAP (at 405nm ex, 515nm em) for respective groups normalized against Os-/Veh (n=3; Transwell co-culture: **=*p*<.01 vs. Os-/Veh; Layered co-culture: *=*p*<.05 vs. Os-/Veh). Os- =basal media, Os+ =osteogenic media, Veh= vehicle, Mel= melatonin, SC= strontium citrate, D3= vitamin D_3_ (Cholecalciferol), K2= vitamin K_2_ (MK7).

The effect of MSDK on TRAP levels was measured since TRAP is an well-known marker for terminally differentiated osteoclasts and their bone resorption activity [25, 26]. Figure 7A demonstrates an MSDK-mediated inhibitory effect on transwell osteoclastic TRAP releasing activity, which was not observed in layered co-culture or when the components were given alone (Fig. 7B). These findings indicate that the direct interaction between osteoblasts and osteoclasts modulate each others' activity—direct contact between human mesenchymal stem cells and peripheral blood monocytes undergoing differentiation into osteoblasts or osteoclasts attenuated MSDK-mediated increases in osteoblasto-genesis and attenuated osteoclastogenesis, respectively.

### MSDK modulates OPG and RANKL levels in co-cultures of hMSCs and hPBMCs dependent upon the type of culturing condition—layered or transwell

Figure 8 illustrates the effect of MSDK on osteo-protegerin (OPG) and receptor activator of nuclear factor kappa-B ligand (RANKL)—signaling proteins known to modulate osteoclast activity and differentiation. As shown in figure 8Ai, MSDK increased the ratio of OPG:RANKL in transwell osteoblasts compared to Os+/Veh; this was due to increases in OPG (Fig. 8Aii) and decreases in RANKL (Fig. 8Aiii). Similar effects were observed when hMSCs were grown as monolayers (Fig. 8Ci and ii); in the absence of hPBMCs except that RANKL protein remained unchanged (Fig. 8Ciii) at levels similar to control. Interestingly, when human mesenchymal stem cells (hMSCs) were cultured in direct contact with peripheral blood monocytes (hPBMCs), no MSDK-mediated increases in the ratio of OPG:RANKL occurred (Fig. 8Bi); no further enhancement of OPG (Fig. 8Bii) or decrease in RANKL (Fig. 8Biii) occurred vs. osteogenic medium containing vehicle (Os+/Veh). The attenuation of osteoclastogenesis in transwell co-cultures vs. layered may be due to the secretory pattern of osteoprotegerin (sOPG) from the osteoblast rather than sRANKL (Figs. 8D, 8E). For example, Os+/Veh and Os+/MSDK in both the transwells (Fig. 8D) and monocultures (Fig. 8E) induced sRANKL compared to Os-/MSDK. High sRANKL (Figs. 8Diii, 8Eiii) under these conditions should increase osteoclastogenesis, but this did not occur as shown in Figure 7. In fact, osteoclastogenesis was inhibited in both co-cultures in the presence of osteogenic medium and, even more so, in the transwell co-cultures containing MSDK. The fact that osteoprotegerin was the only protein modulated by MSDK and that its level correlated with osteoclasto-genesis suggests that secreted osteoprotegerin and not secreted RANKL was modulating osteoclastogenesis. The presence of the osteoclast in the transwell co-culture is probably modulating OPG release from the osteoblast because secreted OPG levels in hMSC monocultures decreased in response to MSDK. Perhaps, the inhibition of osteoclastogenesis observed in the transwell co-cultures was due to OPG-mediated decreases in free RANKL; this would decrease RANK activation on osteoclasts attenuating osteoclastogenesis and activity. Another possibility is that differential processing of RANKL by proteinases located on the osteoblasts creates soluble RANKL products less capable of generating osteoclasts [27]. This is supported in figure 8F demonstrating the formation of different RANKL peptide fragments, particularly the 25kDa (Fig. 8Fi and 8G) and 24kDa fragments (Fig. 8Fii), which indicates ectodomain shedding by a disintegrin and metalloproteinase domain-containing protein (ADAM) 10 or matrix metalloproteinase (MMP) 14 [28].

**Figure 8 A-C:**
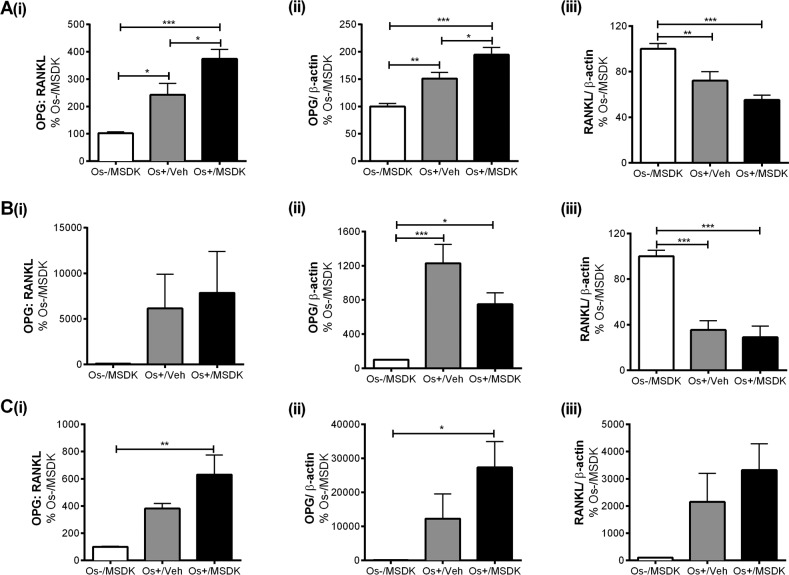
Effect of MSDK on sOPG and sRANKL expression in osteoblasts grown in transwell co-cultures, layered co-cultures and hMSCs monocultures After 21 days of MSDK exposure, OPG and RANKL expression in osteoblasts was measured by western blot in (**A**) transwell co-cultures, (**B**) layered co-cultures and (**C**) hMSCs monocultures. Cell lysates were prepared on day 21 from the bottom (osteoblasts) and top (osteoclasts) chambers in the transwell co-culture and from the whole plate (both osteoblast and osteoclast) in the layered co-culture. Protein levels were normalized against β-actin and then to Os-/MSDK. Mean expression of (**i**) OPG: RANKL, (**ii**) OPG and (**iii**) RANKL in each culture were analyzed, normalized against Os-/MSDK and compared between groups (Os-/MSDK, Os+/Veh, Os+/MSDK).

**Figure 8D-G:**
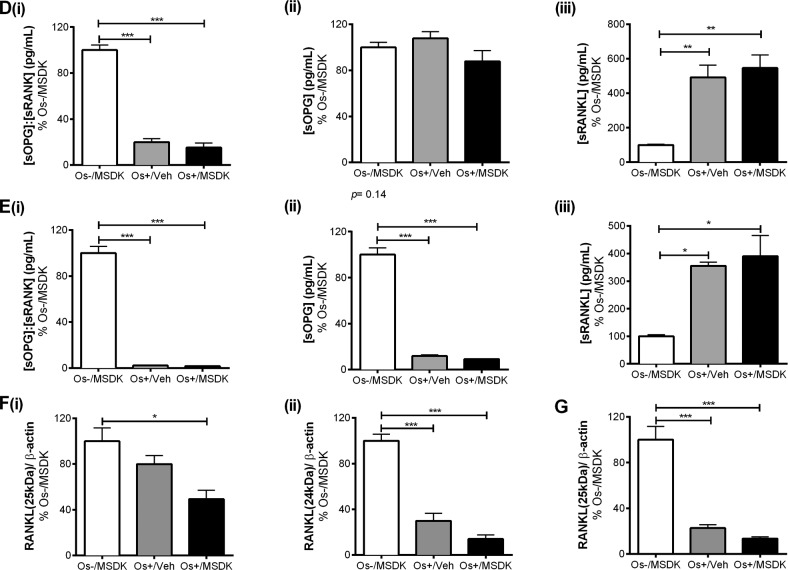
Effect of MSDK on sOPG and sRANKL expression in osteoblasts grown in transwell co-cultures, layered co-cultures and hMSCs monocultures Treatments effects on secreted OPG (sOPG) and RANKL (sRANKL) were measured by ELISA in (**D**) transwells and (**E**) monocultures. Mean concentrations (pg/mL) of (**i**) sOPG: sRANKL, (**ii**) sOPG and (**iii**) sRANKL in culture media were analyzed, normalized against Os-/MSDK and compared between groups. Treatment effects on RANKL processing were detected via western blot by measuring the mean osteoblast's expression of (**i**) 25 KDa and (**ii**) 24 KDa RANKL fragments in (**F**) transwells; and (**G**) 24 KDa RANKL fragment in layered co-cultures. *=*p*<.05, **=*p*<.01 and ***=*p*<.001; One-way ANOVA followed by Bonferroni's post-hoc multiple comparison *t*-test (n=6 per group).

### MSDK modulates pERK1/2 and pERK5 levels in co-cultures of hMSCs and hPBMCs dependent upon the type of culturing condition—layered or transwell

Figures 9A and B illustrate the effect of MSDK on ERK1/2—signaling proteins known to modulate osteoblast differentiation [29–33]. MSDK primarily enhanced ERK1/2 activity (pERK1/2) and down-regulated total ERK1/2 (tERK1/2) in osteoblasts. Similar effects occurred in the layered co-cultures containing both osteoblasts and osteoclasts (Fig. 9B). Previous studies have also shown that ERK5 plays a role in both osteoblast and osteoclast function and differentiation [34–36]. Therefore, MSDK effects on ERK5 in all three-cell culture models—transwell, layered and monolayers—were assessed. Figure 9C illustrates the effect of MSDK on transwell osteoblastic ERK5 expression. As shown, hMSCs exposed to osteogenic (Os+) medium alone had decreased pERK5 activity compared to growth medium containing MSDK (Os-/MSDK), which was not due to decreases in total ERK5 (tERK5) levels. The addition of MSDK to the Os+ media produced an increase in pERK5 relative to Os+/Veh; however, this may be due to decreases in tERK5 since levels decreased when compared to Os-/MSDK. Interestingly, when human mesenchymal stem cells (hMSCs) were cultured in direct contact with peripheral blood monocytes (hPBMCs) (layered), no changes in pERK5 or tERK5 occurred (Fig. 9D). It appears that the presence of the osteoclast exerts an inhibitory influence over osteoblastic pERK5 because when hMSCs were grown in the absence of osteoclasts, MSDK increased pERK5 and tERK5 (Fig. 9E). Another alternative could be that MSDK exerts an opposite effect on pERK5 in periopheral blood monocytes compared to mesenchymal stem cells and because layered co-cultures contain both osteoblasts and osteoclasts, the net effect of MSDK would be zero. This idea is supported in figure 9F demonstrating a trend towards increase in transwell osteoclastic pERK5 in response to MSDK.

**Figure 9A-C:**
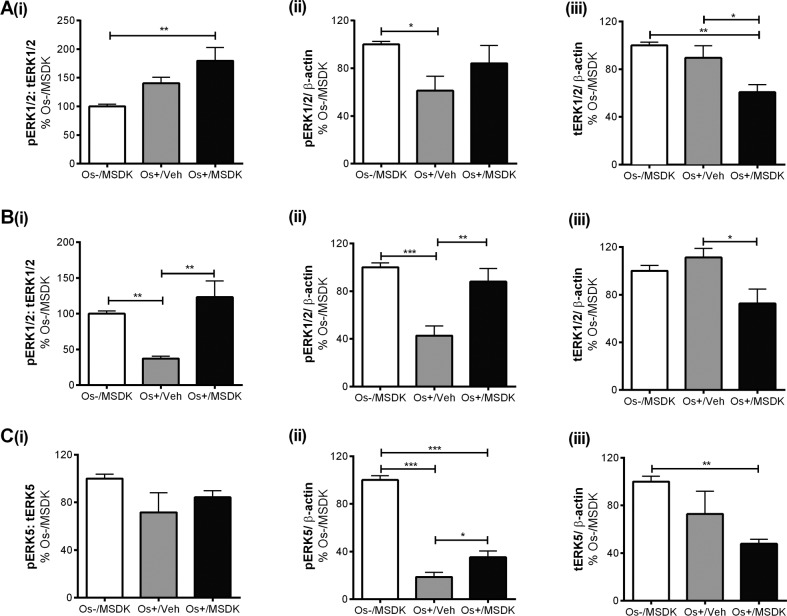
Effect of MSDK on MAPKs, ERK1/2 and ERK5 After 21 days of MSDK exposure, western blot was performed to determine (**A**) ERK1/2 expression of osteoblasts grown as transwell co-cultures, (**B**) ERK1/2 expression of osteoblasts and osteoclasts grown as layered co-cultures, (**C**) ERK5 expression of osteoblasts grown as transwell co-cultures.

**Figure 9D-F:**
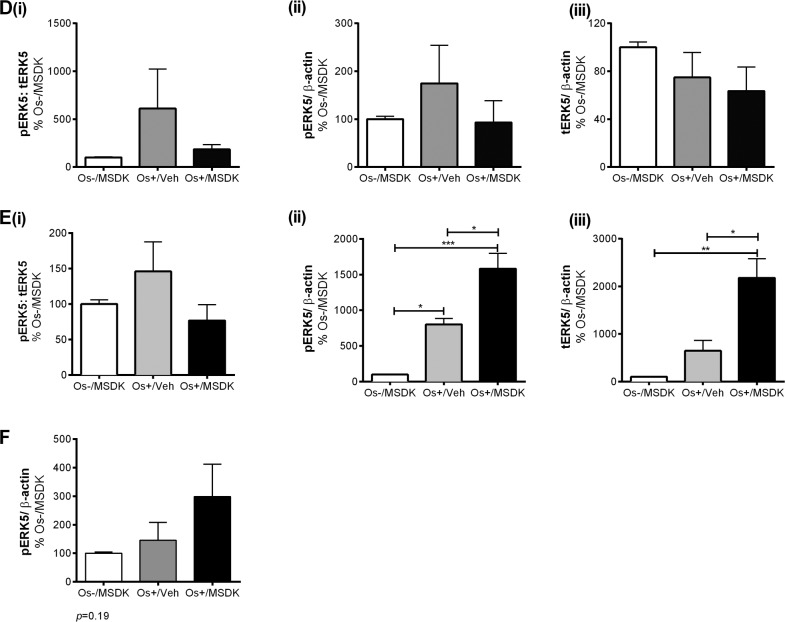
Effect of MSDK on MAPKs, ERK1/2 and ERK5 (**D**) ERK5 expression of osteoblasts and osteoclasts grown in layered co-culture, (**E**) ERK5 expression of osteoblasts in hMSCs mono-culture and (**F**) phospho-ERK5 expression of osteoclasts grown in transwell co-culture. Cell lysates were prepared on day 21 from the bottom (osteoblasts) and top (osteoclasts) chambers in the transwell co-culture and from the whole plate (both osteoblast and osteoclast) in the layered co-culture. Protein levels were normalized against β-actin and then to Os-/MSDK. Mean expression of (**i**) phospho-ERK: total-ERK (**ii**) phospho-ERK (**iii**) total-ERK in each co-cultures were analyzed and compared between groups (Os-/MSDK, Os+/Veh, Os+/MSDK). *=*p*<.05, **=*p*<.01 and **=*p*<.001; One-way ANOVA followed by Bonferroni's post-hoc multiple comparison *t*-test (n=6 per group).

### MSDK modulates RUNX2 levels in co-cultures of hMSCs and hPBMCs dependent upon the type of culturing condition—layered or transwell

Figures 10A and B illustrate the effect of MSDK on Runt-related transcription factor 2 (RUNX2)—the master regulator of osteogenesis [31, 32] regulated by MAPKs [31, 32] and melatonin in osteoblasts differentiated from hMSCs [33] and in bone [37, 38]. Like many of the other proteins studied, the type of co-culture dictated the responses to MSDK. For example, MSDK enhanced RUNX2 expression in transwell osteoblasts beyond that induced by Os+/Veh (Fig. 10A). However, in layered osteoblasts, MSDK did not further enhance RUNX2 expression induced by Os+/Veh (Fig. 10B). The latter may be due to the possibility that maximal levels of RUNX2 expression may already have been attained.

**Figure 10A-F:**
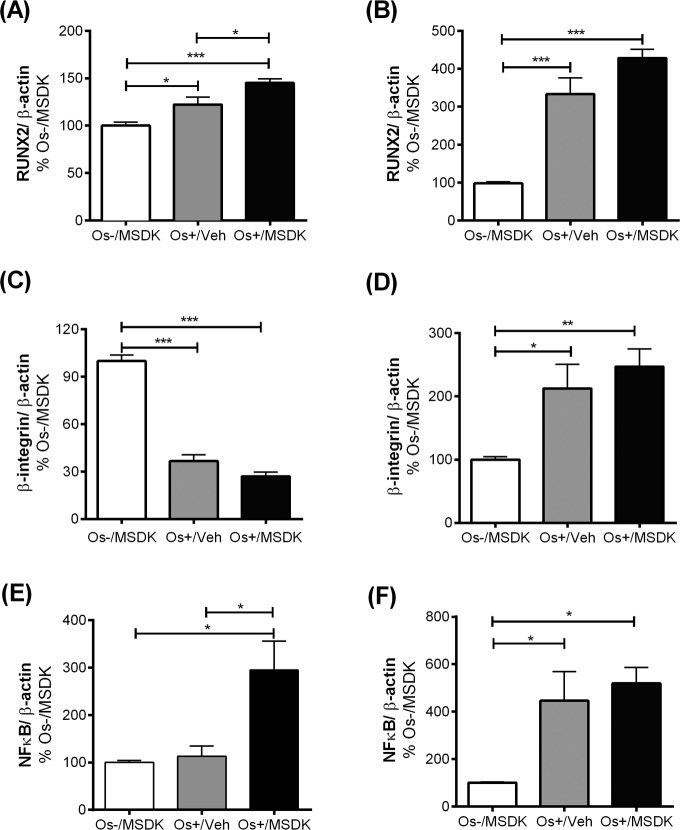
Effect of MSDK on RUNX2, β1 integrin, NFkB and metabolic proteins After 21 days of MSDK treatment, western blot was performed to determine (**A**) RUNX2 expression of osteoblasts grown in transwell co-cultures, (**B**) RUNX2 expression of osteoblasts grown in layered co-culture, (**C**) β1 integrin expression of osteoblasts grown in transwell co-cultures, (**D**) β1 integrin expression of osteoblasts and osteoclasts grown in layered co-cultures (**E**) NFκB expression of osteoclasts grown in transwell co-cultures, and (**F**) NFκB expression of osteoblasts and osteoclasts grown in layered co-cultures.

**Figure 10G-H:**
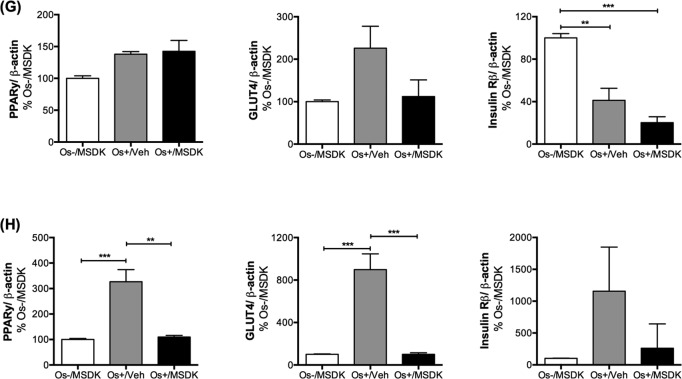
Effect of MSDK on RUNX2, β1 integrin, NFkB and metabolic proteins Metabolic proteins such as PPARγ, GLUT4 and Insulin Rβ expression were also measured in (**G**) osteoblasts grown in transwell co-cultures and (**H**) in osteoblasts and osteoclasts grown in layered co-cultures. Cell lysates were prepared on day 21 from the bottom (osteoblasts) and top (osteoclasts) chambers in the transwell co-culture and from the whole plate (both osteoblast and osteoclast) in the layered co-culture. Protein levels were normalized against β-actin and then to Os-/MSDK. Mean protein levels were analyzed and compared between groups (Os-/MSDK, Os+/Veh, Os+/MSDK). *=*p*<.05, **=*p*<.01 and **=*p*<.001; One-way ANOVA followed by Bonferroni's post-hoc multiple comparison *t*-test (n=6 per group).

### MSDK did not modulate β1 integrin level in co-cultures of hMSCs and hPBMCs

Figures 10C and D illustrate the effect of MSDK on β1integrin—proteins demonstrated to regulate the interaction between bone cells and extracellular matrix and thus control different aspects of bone cell growth and activity [39]. The use of these two different co-culture models permitted exploration of the roles of MSDK on cell matrix proteins especially in layered co-cultures where osteoblasts and osteoclasts are in direct contact with each other during the differentiation process. As shown in figures 10C and 10D, the effect of osteogenic (Os+) medium alone on β1 integrin expression was opposite in effect depending on the type of co-culture—osteogenic medium alone (Os+/Veh) induced osteoblastic β1 integrin levels in the layered co-culture (Fig. 10D) but decreased its levels in transwell osteoblasts (Fig. 10C). The addition of MSDK to the osteogenic medium did not further increase or decrease β1 integrin levels in either co-culture.

### MSDK modulates NFkB levels in co-cultures of hMSCs and hPBMCs dependent upon the type of culturing condition—layered or transwell

Figure 10E demonstrates the effect of MSDK on nuclear factor-κB (NFκB)—a protein shown to play a vital role in RANK-mediated osteoclastogenesis [40–42] and osteoblastogenesis [43, 44]. As shown in figure 10E, peripheral blood monocytes (hPBMCs) grown as transwell co-cultures with mesenchymal stem cells (hMSCs) exposed to osteogenic (Os+) medium con-taining MSDK demonstrated significant increases in NFκB compared to growth medium containing MSDK (Os-/MSDK) or osteogenic medium containing vehicle (Os+/Veh). No increases in NFκB occurred in the presence of osteogenic medium (Os+/Veh) alone. In contrast, exposure to osteogenic medium alone (Os+/Veh) increased NFκB levels in layered co-cultures but no further enhancement occurred in presence of MSDK (Fig. 10F). The effects of Os+/MSDK on NFκB levels in transwell osteoclasts are not easily explained considering that this same culture condition (transwell and Os+/MSDK) decreased osteoclastogenesis (Fig. 7A).

### MSDK modulates PPARγ and GLUT4 levels in co-cultures of hMSCs and hPBMCs dependent upon the type of culturing condition—layered or transwell

Figure 10G and H illustrate the effect of MSDK on peroxisome proliferator-activated receptor gamma (PPARγ), glucose transporter type 4 (GLUT4) and the beta subunit of insulin receptor (Insulin Rβ)—metabolic proteins shown to modulate bone cell differentiation and activity [45–48]. Again, the type of culturing condition played vital roles in their expression in osteoblasts exposed to MSDK. For example, MSDK inhibited PPARγ and GLUT4 levels in layered osteoblasts compared to cells exposed to osteogenic medium alone (Fig. 10H), but not in transwell osteoblasts (Fig. 10G). Insulin Rβ levels, though, were only modulated in transwell osteoblasts exposed to osteogenic medium alone (Fig. 10G). No effect on Insulin Rβ levels occurred in layered osteoblasts in response to any of the treatments (Fig. 10H).

### MSDK induces osteoblastogenesis in hAMSCs

**Figure 11:**
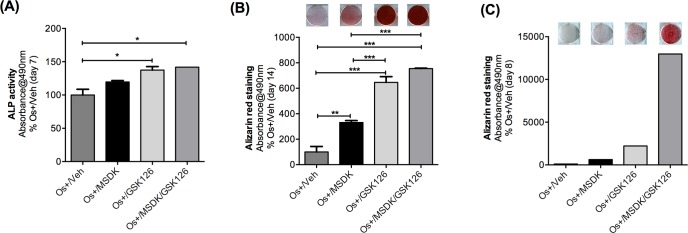
Effect of MSDK on adipose-derived mesenchymal stem cells Adipose-derived mesenchymal stem cells (AMSCs) were exposed to MSDK for 7 days (**A**), 8 days (**C**) or 14 days (**B**) to assess its effects on alkaline phosphatase (**A**) or alizarin red staining (**B, C**). Red color indicates calcium deposition by MSCs. Each bar represents the mean absorbance of alkaline phosphatase activity or alizarin red at 490nm for respective groups Os+/Veh; Os+/GSK126; Os+/MSDK; Os+/GSK126/MSDK repeated twice in triplicate (except for the alizarin red experiment at day 8). GSK 126 = S-adenosyl-methionine-competitive small molecule inhibitor of EZH2 methyltransferase is known to promote osteogenic differentiation of MSCs through effects on the osteoblast epigenome [49, 50]. The observed co-stimulatory effects of MSDK and GSK126 suggest mechanistic synergy.

Because MSDK produced important changes in PPARγ and GLUT4—proteins known to play important roles in the switch from adipogenesis to osteogenesis—MSDK effects on adipose-derived mesenchymal stem cells (MSCs) were assessed. As shown in figure 11, MSDK in combination with osteogenic medium (OS+/MSDK) strongly induced osteoblast differentiation of human adipose-derived mesenchymal stem cells reflected by increases in alkaline phosphatase levels at day 7 (Fig. 11A), and increases in alizarin red staining at day 8 (Fig. 11C), which peaked by day 14 (Fig. 11B). Interestingly, we observed that MSDK effects were strongly enhanced in the presence of GSK126, an inhibitor of EZH2, a lysine methyl transferase. Because EZH2 is known to suppress osteogenic differentiation of MSCs and inhibit maturation of osteoblasts limiting bone accrual *in vivo* [49, 50], the MSDK regimen mechanistically synergizes with EZH2-mediated epigenetic control of bone cell differentiation.

## DISCUSSION

Women begin to experience bone loss during late perimenopause, which continues even after menopause. In fact, half of the bone loss observed in women during the course of their lifetime occurs during the first 10 years postmenopause [51]. Our study is one of the first addressing the need for starting bone intervention therapies earlier to reverse bone loss in an osteopenic population. This is important because although the recurrent fracture rates are high in osteoporosis, new fractures predominantly arise in the osteopenic population [4, 10]. Therefore, this shift to earlier intervention starting with osteopenia rather than osteoporosis may lead to the prevention of most fractures observed worldwide. In our study, osteopenic women taking MSDK for one year had a significant improvement in their left femoral neck and lumbar spine BMD and demonstrated a lower risk for a major osteoporotic fracture risk compared to women taking placebo. This is consistent with previous studies that demonstrate an increased BMD using melatonin alone [12], strontium alone [13, 14] or combination vitamin D_3_ and vitamin K_2_ [17, 18] or combination strontium citrate, vitamins K_2_ and D_3_ [20]. MSDK was found to be less effective in improving hip BMD, perhaps due to the kinetics of bone remodeling in long vs. flat bones [52]. Concomitant with the increases in BMD were increases in serum P1NP levels resulting in decreases in bone marker turnover (↓CTx:P1NP)—this was not observed in clinical studies using melatonin. In the Treatment of Osteopenia with Melatonin (MelaOst) study, one year dosing with melatonin (1mg, 3mg) demonstrated positive effects on femoral neck bone density in postmenopausal women with osteopenia [12, 53]; however, no changes in any of the serum bone markers tested occurred. These findings from the MelaOst study, though important, suggest that the mechanism for melatonin-induced increases in BMD may be through calcium-mediated bone mineralization, which may produce a ceiling effect of melatonin alone on improving bone health if taken for long periods of time. Also, the higher dose of melatonin in the MOTS may be producing other beneficial effects on bone through melatonin's anti-oxidant properties. The changes in serum P1NP is most likely due to strontium (citrate) component of MSDK since strontium (ranelate) in The Spinal Osteoporosis Therapeutic Intervention (SOTI) study increased bone-specific alkaline phosphatase (BSAP) by 8.1% [14]. In the MOTS, decreases in the ratio of bone resorption to bone formation (i.e., ↓CTx:P1NP) suggest that MSDK may be renormalizing bone marker turnover towards equilibrium resulting in increases in BMD and, if taken for extended periods of time, may continue to reverse bone loss through the aging process. Because P1NP is a marker of bone formation, the MSDK-mediated increases in P1NP may reflect increases in osteoblast differentiation. Although seasonal variation of vitamin D3 occurs [71] which could impact on serum CTx levels [23], both vitamin D3 and CTx levels remained similar between groups at all timepoints despite the fact that we enrolled and assessed participants' serum vitamin D3 and CTx levels between August 2013 to April 2015.

This concept is supported in the human osteoblast and osteoclast co-culture studies—both transwell and layered—demonstrating that combination MSDK induced osteoblastogenesis to a much greater extent than that induced by melatonin or strontium (citrate) alone and that only combination MSDK inhibited osteoclastogenesis. The differences in the robustness of MSDK's effects on osteoblast and osteoclast differentiation (i.e., greater increase in transwell osteoblast differentiation vs. layered; greater decrease in layered osteoclast differentiation vs. transwell), could be explained by the types of signaling pathways activated (pERK1/2, pERK5 and RUNX2) in transwell osteoblasts. This interpretation is supported by the findings that MSDK exposure induced transwell osteoblast pERK1/2, pERK5, and RUNX2 expression. Based on previous studies [29, 33] and the MOTS, we propose that MSDK, through ERK1/2/5-mediated increases in RUNX2 expression in transwell human mesenchymal stem cells, increases their differentiation into osteoblasts. Because other pathways, such as p38, JNK, BMPs, canonical WNT also regulate RUNX2 transcriptional activity [54–56], these pathways cannot be ruled out and require further investigation.

MSDK treatment triggered osteoblasts to produce more osteoprotegerin and less RANKL in the transwell co-cultures. The relative expression of osteoprotegerin and RANKL in osteoblasts is a critical transition point for balancing bone mineralization [41]. MSDK is more than likely balancing osteoblast and osteoclast activities, initially, through its differentiating effects on osteoblasts and then begins to modulate RANKL-mediated osteoclastogenesis through osteoblast-mediated osteoprotegerin expression to maintain appropriate bone remodeling. This concept is corroborated in the MOTS demonstrating a steady level of CTx throughout MSDK treatment. Besides being reduced as osteoblasts mature, RANKL can go through various stages of processing to regulate osteoclast activation; this processing is generated from the ectodomain shedding of membrane-bound RANKL via the action of MMP 3, 7 and 14 and ADAM 10, 17 (or TACE) and 19 [27, 42]. In our study, MSDK, in combination with osteogenic (Os+) medium, decreased the 25kDa RANKL peptide fragment in transwell co-cultures indicative of a facilitation of ADAM 10's catalytic activity [28]. In addition to MSDK's effect on osteoblasts, osteoclast-mediated modulation of osteoblast differentiation may be occurring through paracrine factors released by osteoclasts (e.g., IGF1 and IGF2, FGF2, TGFB1 and TGFB2, BMP2, -3, -4, -6 and -7, PDGF) to favor osteoblast formation in transwells or through the osteoblast-osteoclast contact-dependent ephrin-signaling pathway [57–59].

Bone loss therapies often fail to produce their desired effect because of poor compliance and limited adherence to the treatments. One study showed that the compliance rate for taking osteoporosis medications was <80%, as measured by the medication possession ratio (MPR), and this was associated with a 17% increase in fracture rate [60]. In our study, MSDK resulted in high (92%) compliance. Two of the participants dropped out from the study—one from the placebo group and another from the MSDK group due to general illness. This is in contrast to the Combination of Micronutrients for Bone (COMB) study, which had lower compliance—about 37 out of 114 patients (32.45%) in the COMB did not comply with the therapy [20]. The cohort differences between COMB and MOTS were study size (n=114 vs. n=20 respectively) and the baseline bone density status (osteoporosis vs. osteopenia respectively). Higher doses of strontium (citrate; 680 mg) and vitamin K_2_ (100 mcg) were used in COMB compared to our study, which used 450 mg of strontium (citrate) and 60 mcg of vitamin K_2_. Also unique to our study is the inclusion of melatonin, which has been shown to restore bone marker balance [11]. Melatonin is an efficacious agent for entraining sleep rhythms and this fact could improve compliance due to the positive reinforcing effects of melatonin on sleep quality and mood. This is supported in the MOTS, in which 29% more positive/neutral comments about sleep and 14% more positive comments about mood were made in the MSDK group vs. placebo. Besides melatonin, the strontium (citrate) component in MSDK may be contributing to improvements of quality of life. In the SOTI trial, quality of life slightly improved using QualiOst which persisted for up to 4 years as reviewed [61]. Besides affecting compliance, melatonin's ability to regulate sleep rhythms may be improving bone health. In postmenopausal women, poor sleep quality, specifically going to bed at a later bedtime, sleeping late into the morning and frequent daytime napping was associated with low BMD [62]. The melatonin component in MSDK may be keeping bone rhythms synchronized with the light/dark cycle preventing osteopenia and osteoporosis.

As of today, no data are available regarding an appropriate therapeutic range for melatonin to produce positive effects on bone. Women taking the MSDK supplements (which contained 5mg melatonin) had ∼140 times more nocturnal melatonin than women taking placebo. In fact women taking placebo had very low endogenous nocturnal urinary melatonin-sulfate levels consistent with reports showing low melatonin levels in an aged and menopausal population [63, 64]. Even though women in the MSDK group took supplements at night for a year, their urinary melatonin-sulfate levels varied, possibly due to the differences in bioavailability similar to what was seen in males (range: 10%-56% in men; mean 33%) [65]. This study observed a direct relationship between urinary melatonin-sulfate levels and lumbar spine BMD similar to the findings of Amstrup and colleagues showing a dose-dependent effect of melatonin on femoral neck BMD [12]. Therefore, exogenous melatonin supplementation could play an important role in maintaining bone density in postmenopausal osteopenic women.

After one year of MSDK treatment, CRP levels of the participants dropped below 1.4mg/L with a mean group value of 0.57mg/L compared to the placebo mean value of 1.5mg/L. A reduction in CRP levels by MSDK could imply a possible anti-inflammatory role of MSDK, aiding in the bone health and other diseases such as cardiovascular and metabolic disorders as reported [66–70]. Moreover, MSDK treatment did not have any worsening effect on blood pressure, indicating its relative safety in postmenopausal population in terms of cardiovascular events.

Morphometric analyses were conducted to determine if any changes in body morphometry occurred in response to MSDK. Although no significant changes occurred in response to MSDK treatment when compared to placebo, there was less weight variance throughout the year in the MSDK group compared to placebo group (*p*= 0.032). Perhaps MSDK, most likely through melatonin, works to stabilize weight fluctuation, which could provide some bone protection. Dramatic changes in weight can contribute to increases in bone turnover and decreases in bone mass [24]; and mature women with a BMI lower than 18kg/m^2^ are estimated to have more than 30% bone loss than normal women of same age [72]. In the MelaOst trial, postmenopausal osteopenic women taking melatonin had a decrease in total fat mass and trended towards an increase in lean body mass [53]. This shifting away from adipogenesis towards osteogenesis may explain, in part, MSDK's actions on bone, which is supported in the co-cultures studies using human mesenchymal stem cells and peripheral blood monocytes but also in the adipocyte derived mesenchymal stem cells.

PPARγ, although a key regulator in adipogenesis, energy expenditure and lipid, glucose, and insulin metabolism, produces undesirable effects on bone. High levels of PPARγ may cause bone loss by switching the fate of mesenchymal stem cells towards adipogenesis rather than osteoblastogenesis [45] resulting in increased fat content in the bone or increased RANKL production [45, 73]; low levels of PPARγ increase bone mass by enhancing osteoblastogenesis [45, 47, 74]. In layered osteoblasts, MSDK inhibited both PPARγ and GLUT4 expression induced by osteogenic medium in layered osteoblasts. These findings for PPARγ are consistent with other studies using melatonin and strontium (ranelate) on osteoblastogenesis [75, 76]. Correlations between PPARγ and GLUT4 expression have been observed in other studies [77, 78]. The findings that melatonin improved femoral bone mineral density [12] and decreased total fat mass [53] in postmenopausal osteopenic women and that MSDK increased BMD and demonstrated a reduction in weight fluctuation in a similar cohort suggests that MSDK may be inducing new bone formation by modulating the metabolic parameters in human mesenchymal stem cells. Specifically, MSDK may be driving mesenchymal stem cells down to osteogenic rather than adipogenic lineage through the downregulation of PPARγ and GLUT4 in adipose-derived mesenchymal stem cells where MSDK induced their differentiation into osteoblasts. This is also supported by our findings in adipocytes-derived mesenchymal stem cells where MSDK induced their differentiation into osteoblasts.

**Figure 12:**
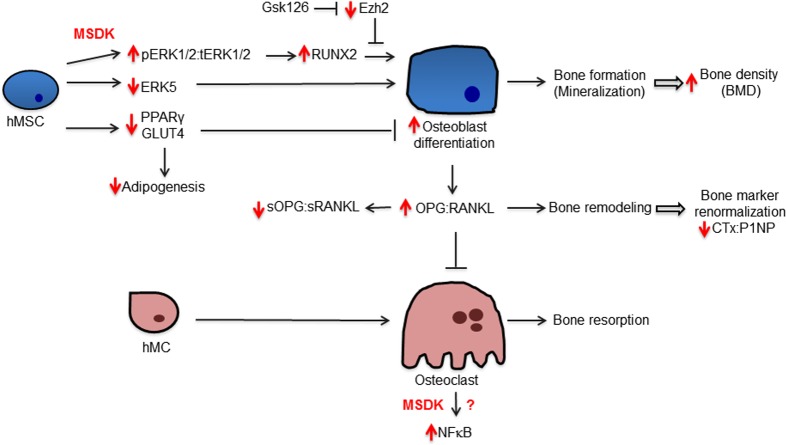
Potential mechanism underlying MSDK effects on bone formation The diagram illustrates regulatory and biological relationships between the indicated proteins and treatments.

In conclusion, combination melatonin, strontium (citrate), vitamin D_3_ and vitamin K_2_ significantly increased lumbar spine BMD by 4.3% and left femoral neck BMD by 2.2%, with a trend (p=0.069) towards an increase in hip BMD from baseline after one year in postmenopausal osteopenic women. The 10-year vertebral fracture risk probability decreased by 6.48% in response to MSDK therapy compared to 10.8% increase in placebo. MSDK reduced bone marker turnover primarily by increasing the bone formation marker P1NP and maintaining healthy bone turnover (↓CTx:P1NP ratios). MSDK demonstrated positive effects on inflammatory status and improved quality of life especially related to sleep. MSDK did not produce adverse effects psychologically as well as physically and did not negatively affect compliance rate. MSDK may be modulating osteoblast and osteoclast function via the release of paracrine factors, osteoprotegerin and RANKL from osteoblasts. These studies also describe novel signaling cascades (Fig. 12) underlying MSDK effects on osteoblastogenesis and osteoclastogenesis that include pERK1/2, pERK5, RUNX2, ADAMs, PPARγ and GLUT4. Importantly, the mechanism of action of MSDK cocktail synergizes with the osteogenic effects obtained from inhibiting the epigenetic regulator EZH2, which normally suppresses osteogenic differentiation *in vitro* and bone accrual *in vivo* [49, 50]. Because EZH2 inhibition similar to MSDK treatment has favorable bone anabolic outcomes *in vivo*, the possibility arises that combination treatment of MSDK with EZH2 inhibitors (e.g., GSK126) could be beneficial at least for short-term therapies aimed at increasing bone mineral density.

Based on the fact that MSDK treatment did not completely inhibit osteoclastogenesis, we conclude that MSDK is favoring bone remodeling to proceed towards equilibrium by allowing osteoclastogenesis to some extent. These *in vitro* studies support our MOTS clinical trial findings demonstrating that MSDK reduces bone turnover rate by increasing P1NP expression, while maintaining steady levels of CTx. Results from this study underscore the complexity but therapeutically relevant effects of MSDK on bone cell development and activity making MSDK a viable and potential alternative therapy for managing and/or treating osteopenia in postmenopausal women. Large-scale, multicenter randomized control trials testing the efficacy of MSDK to prevent and treat osteopenia are warranted. Limitations to this study include low number of subjects, lack of a diverse cohort and lack of different micronutrient combinations on primary and secondary endpoints in MOTS clinical trial.

## METHODS

### Participant screening and recruitment

A randomized double-blind placebo controlled one-year study was designed. Investigation has been conducted in accordance with the ethical standards and according to the Declaration of Helsinki and national and international guidelines; and has been approved by the Duquesne University Institutional Review Board (ClinicalTrials.gov Identifier: NCT01870115 and IRB Grant protocol number 13-59). Inclusion criteria included being postmenopausal with osteopenia (T-score between −1 to −2.5); willingness to participate in a 12-month study; willingness to take daily therapy right before bed; willingness to undergo testing of bone markers and other biochemical parameters; and to provide a self-assessment on quality of life throughout the program. Exclusion criteria included being diagnosed with osteoporosis (T-score less than −2.5); being osteopenic as a consequence of other medical conditions such as hyperparathyroidism, metastatic bone disease, multiple myeloma or chronic steroid use; current use of hormone therapy or birth control; current use of prescription medications for osteoporosis, sleep, depression, anxiety, ulcerative colitis or regulation of blood pressure; current use of steroid medications or chronic use in the past 6 months; other medical conditions such as uncontrolled high blood pressure, liver disease, severe sleep apnea, chronic obstructive pulmonary disease; and current use of tobacco. Individuals who satisfied the eligibility criteria were invited to schedule an initial visit to the Center for Pharmacy Care at Duquesne University. At the first visit, participants' osteopenia was confirmed by DXA. Blood pressure readings were taken from both arms to ensure that participants had an average blood pressure between 140/90 and 100/60. Women who fulfilled the bone density and blood pressure requirements were invited to enroll in the study. Informed consent was obtained from all study participants. During the first visit, subjects completed forms detailing basic demographic information and listed all prescription and nonprescription drugs and/or supplement usage.

### Randomization and follow-up

The recruited postmenopausal women were randomly assigned to receive either placebo (n=11) or MSDK (n=11) treatments based on a computer-generated block randomization scheme. Allocation concealment was maintained among the participants and principal investigators throughout the randomization and throughout the study. Treatment capsules, referred to as “MSDK” were formulated using 5 mg melatonin, 450 mg strontium (citrate), 2000 IU vitamin D3 and 60μg vitamin K2, divided into two capsules. Identical placebo capsules matching in size, shape and color contained plant fiber. Study capsules were formulated and manufactured according to the principal investigators' specifications and supplied by Pure Encapsulations, Inc. (Sudbury, MA, USA). Subjects were instructed to take two capsules by mouth daily at their usual bedtime. After the first visit, bimonthly visits (at months 2, 4, 6, 8, 10 and 12) were arranged at the Center for Pharmacy Care (Duquesne University) over a 12-month period. During each visit, a registered nurse blinded to the group assignments performed the study-related health assessments. Participants received a 2-month supply of medication and daily diaries at each visit to keep track of their daily pill intake, sleep duration, physical activity and any other information that the participant would feel was important to note about their general well-being. In addition to study medication, participants were allowed to take <1000 IU of vitamin D3 and <1000 mg of calcium daily.

### Bone density

Bone mineral density (BMD) was measured at baseline and at month 12 in the left femoral neck, total left hip and lumbar spine L1-L4 via Dual-energy X-ray absorptiometry (DXA). Mean changes in bone density (BMD) from baseline to month 12 were evaluated and compared between groups.

### Bone turnover rate

Bone marker turnover was assessed in serum by collecting blood samples at months 0, 6 and 12. Serum was stored at −20°C until the time of analysis. All samples were tested at a similar time point to minimize any analytical variation. Bone formation markers total procollagen type 1 amino-terminal propeptide (P1NP) and osteocalcin (OC; both intact and N-terminal mid-fragments) were measured in the serum samples via ELISA using the human total P1NP ELISA kit for serum (Mybiosource, CA, USA) and osteocalcin (1-43/49) enzyme immunoassay assay kit for serum (ALPCO Diagnostic, NH, USA), respectively, per kit instructions. Bone resorption marker, collagen type I c-telopeptide (CTx), was measured via ELISA using Human Cross-linked Carboxy-terminal Telopeptide Of Type I Collagen (CTX-I) ELISA kit for serum (Mybiosource, CA, USA) following kit instructions. Mean concentration changes in bone markers P1NP (in pg/mL), OC (in ng/mL) and CTx (in ng/mL) were calculated for each time point and compared within and between groups. Ratios of bone resorption: bone formation (CTx:P1NP and CTx:OC) were calculated over time and compared within and between groups. All controls contained within each of the kits were within normal ranges.

### Fracture risk

Fracture risk was assessed at months 0 and 12 using the FRAX^®^–a computer-generated algorithm (http://www.shef.ac.uk/FRAX/) that estimates 10 year probability of a major osteoporotic fracture and a hip fracture in men and women based on their current femoral neck bone density, body composition, previous fractures, parental history of hip fracture and current risk factors [79, 80]. Information required calculating FRAX^®^ scores were collected at months 0 and 12. Mean percent changes in fracture risk from month 0 to month 12 were calculated and compared between groups.

### Vitamin D_3_, C-reactive protein (CRP) and melatonin level

Serum vitamin D_3_ and CRP levels were measured at months 0 and 12 by ELISA using 25(OH) Vitamin D ELISA kit (Enzo Life Science, NY, USA) and High sensitivity human C-reactive protein (hsCRP) ELISA kit (Biomatik LLC. DE, USA), respectively, according to the manufacturer's instructions. Nocturnal urinary melatonin-sulfate levels were measured at the end of the study via ELISA using the Melatonin-Sulfate Urine ELISA kit (IBL International, Germany), following kit instructions. Participants were asked to collect all of their urine between 10pm to 6am throughout the night preceding the day of their last visit. Means ± S.E.M. of vitamin D_3_ (in ng/mL), CRP (in ng/mL) and melatonin (in ng/mL/hr) were calculated for each time point and compared between groups.

### Blood pressure and body composition

Participants' blood pressure was measured at the initial visit and then every two months until the end of the study. Systolic and diastolic blood pressure measurements were taken on three separate occasions from right and left arms and average blood pressure readings for each time point were reported. Morphometric analysis was carried out at months 0 and 12 to assess the treatment effect on body composition. Participants' height, weight, body mass index (BMI), basal metabolic rate (BMR), fat percentage, fat mass (FM), lean body mass (FFM) and total body water (TBW) were measured using TANITA^TM^ body composition analyzer. Mean changes in blood pressure at each time point and mean changes in body composition from month 0 to month 12 were calculated and compared within and between groups.

### Psychometric analyses

Treatment effects on our participants' menopause quality of life, perception of anxiety, non-specific stress and clinical depression states were assessed at months 0, 6 and 12 using four validated questionnaires: MENQOL [81, 82], Spielberger's State-Trait Anxiety Inventory (STAI) [83, 84], Cohen's Perceived Stress Scale (PSS) [84–86] and the Center for Epidemiologic Studies Depression (CES-D) Scale [87], respectively. Questionnaires were administered to the study cohort at months 0, 6 and 12. MENQOL, PSS, STAI and CES-D scores were calculated for each time point; compared within and between groups and reported as mean ± S.E.M.

A daily diary appropriate for a postmenopausal cohort was prepared by modifying the daily diary used in our previous MOPS clinical trial [11]. Participants were asked to record daily information regarding their sleep quality and duration, use of any prescription and non-prescription medication, exercise, and general well-being. Diary pages were collected at each visit and then collated at the end of the study. General well-being was assessed by stratifying the comments into four categories: sleep quality, general mood, GI symptoms and general aches and pains and then sub-stratifying into positive, negative or neutral comments. Comments related to improvement in physical condition or positive feelings were classified as positive comments, whereas comments related to worsening of any existing condition or emerging of a new problem, as well as negative feelings fell under the negative category. Comments made about their daily activities, weather etc., which reflected indifferent thoughts, were classified as neutral comments. For data analyses, the positive and neutral comments made in each cohort were combined per category and then compared to the negative comments made for that same category (placebo vs. MSDK). In addition to their daily logs, compliance was assessed by counting the number of pills remaining from the last visit. Safety and tolerability were assessed at each visit through regular physical examinations and from the daily logs. Treatment effects on sleep duration and exercise intensity were calculated off of the participants' diary log. Exercise intensity was determined following the US Centers for Disease Control and Prevention (CDC) guideline [88]. Based on exercise type and intensity, participants fell into four different groups: no exercise=0; light exercise=1; moderate exercise=2; and high intensity or vigorous exercise=3. Sleep hours and exercise scores were compared between groups and reported as mean ± S.E.M. Use of multivitamins/herbal/OTC supplements were also documented using the diary logs and compared between groups.

### *In-vitro* sample preparation and osteoblast:osteoclast co-cultures

*In vitro* MSDK treatment concentrations were calculated based on the doses used in the MOTS clinical trial. Hence, 50 nM melatonin (M), 191.5 μM strontium citrate (SC), 26 nM vitamin D_3_ (D) and 18.5 nM vitamin K_2_ (K) were dissolved into 100% pure ethanol. Human adult mesenchymal stem cells (hMSCs) (Lonza, MD, USA) were maintained at 37°C, 5% CO_2_ and 90% humidity. Cells were grown and passaged at 80% confluence in mesenchymal stem basal cell growth medium (Os-) (Lonza, USA). Cells were then seeded (at passage 3-5) at an initial density of 3 × 10^3^ cells/cm^2^ in the bottom chamber of transwell plates or in 6-well cell culture plates (Corning, NY, USA), treated with either basal growth media (Os-) or osteogenic media (Os+) (Lonza, USA) with or without treatments (Mel, SC, D3 and K2 either alone or in combination as MSDK) for 21 days. Ethanol (100%) only was used as vehicle (Veh) control. Ascorbate, dexamethasone and β-glycerophosphate present in the osteogenic media (Os+) induced the differentiation of hMSCs into osteoblasts [89]. Full media change occurred once every 3 days. On day 13, blood was taken from healthy consenting female volunteers unrelated to the MOTS clinical trial by the MOTS nurse via venous puncture. Human peripheral mononuclear cells were freshly collected from blood using Ficoll-Paque^TM^ Plus (Amersham Pharmacia Biotech, Sweden) followed by two repeated centrifugations at 400xg for 30-40 min at 18-20°C without break. Monocytes were collected from cells by suspending in RoboSep^TM^ buffer (Stemcell technologies, USA) and using the Easysep™ negative selection human monocyte enrichment kit without CD16 depletion (Stemcell technologies, USA) and purple Easysep™ magnet (Stemcell technologies, USA), following manufacturer's instructions. Monocytes were then seeded (5 × 10^3^ cells/cm^2^) in the top chamber of transwell plate to initiate the transwell co-culture or applied directly on top of the hMSCs to initiate the layered co-culture. Full media changes resumed and occurred once every 3 days until day 21. Parallel studies using hMSCs monocultures were run to determine the influence of osteoclasts on MSDK-mediated osteoblast differentiation. Similar hMSCs plating densities and treatment paradigms were used with the exception being that no hPBMCs were added on day 13.

### Osteoblast differentiation and mineralization

On day 21, calcium mineralization was measured via alizarin red staining assay—this time period was chosen based on past published studies using melatonin to induce differentiation of hMSCs into osteoblasts [33]. Human PBMCs were added to osteoblastic cultures on day 13 because past studies using osteogenic medium have shown that pre-osteoblasts proliferate and start to differentiate into mature osteoblasts between 14 to 21 days [33]; and RANKL, M-CSF and/or OPG levels would be high enough to modulate osteoclastogenesis [41, 90]. Alizarin red staining was performed on the bottom chamber of the transwell co-culture and directly on 6-well plates (both hMSCs and hPBMCs) of the layered co-culture using the commercially available osteogenesis quantification kit (EMD Millipore, MA, USA) per manufacturer's instructions. Microscopic evaluation of osteogenesis was performed using a Vistavision microscope (VWR International) with a progress C3 camera (Zenoptik; Data not shown). Osteogenic differen-tiation was quantified by spectrophotometry at 405 nm using the Perkin Elmer Victor 1420 Multilabel plate reader (Waltham, MA, USA). Concentrations of alizarin red reflecting osteoblast mineralization activity were calculated, normalized against Os-/Veh and compared between groups.

### Osteoclast differentiation and activity

On day 21, tartrate resistant acid phosphatase (TRAP) assays were carried out on the top chamber of transwell co-culture and directly on the 6-well plate (layered co-culture) to measure osteoclastic differentiation and TRAP releasing activity. Quantitative analysis of total TRAP was performed as described by Janckila *et al*., with modification [91]. Briefly, Naphthol-ASBI phosphate (N-ASBI-P) was used as a substrate for TRAP. TRAP buffer was prepared by dissolving N-ASBI-P (2.5mM) in a solution containing 1% ethylene glycol monomethyl ether (EGME), 2% NP40, Na-acetate (100mM) and Na-tartrate (50mM) with pH adjusted at 5.5-6.1. Cells were lysed with 50mM TRIS and treated with TRAP buffer (1mL/well). Cells were then scraped into a 5mL tube along with the buffer and incubated at 37°C for 30 min. Reactions were stopped upon the addition of 2.5 ml 0.1 M NaOH containing 0.05% NP-40. Fluorescence readings were taken at 405nm excitation and 515nm emission wavelength using a Perkin Elmer Victor 1420 Multilabel plate reader. Data were normalized against Os-/Veh and compared between groups.

Qualitative analysis of TRAP was performed directly over the cells attached to plates using the commercially available Acid Phosphatase Leukocyte assay kit (Sigma, USA) per manufacturer's instructions. The blue nuclei were visualized using a Vistavision microscope (VWR international) with progress C3 camera (Zenoptik; Data not shown). Purple staining indicates TRAP deposition by osteoclasts where the amount of TRAP deposition is proportional to the differentiation of osteoclasts.

### Cell lysate preparation and western blotting

On day 21, osteoblast or osteoclast lysates were prepared from the bottom and top chambers of the transwell co-cultures, respectively, or from layered co-cultures containing both osteoblasts and osteoclasts by scraping into Laemmli sample buffer containing β-mercaptoethanol (19:1 ratio), heated for 5 min at 95°C, cooled and then stored at −20°C until use.

Western blotting was performed to measure protein expression of osteoprotegerin (OPG), receptor activator of nuclear factor kappa-B ligand (RANKL), extracellular signal-regulated protein kinases 1 and 2 (ERK1/MAPK3 and MAPK1/ERK2), extracellular-signal-regulated kinase 5 (ERK5/MAPK7), runt-related transcription factor 2 (RUNX2), integrin β1 (ITGB1), nuclear factor kappa B (NFκB), peroxisome proliferator-activated receptors gamma (PPARγ), glucose transporter type 4 (GLUT4/SLC2A4) and beta subunit of insulin receptor (Insulin Rβ) using the Odyssey® Western Blotting Kit IV RD (Licor bioscience, USA). Primary antibodies included rabbit anti-OPG/TNFRSF11B (ab73400, Abcam, USA), rabbit anti-RANKL/TNFSF11 (ab9957, Abcam, USA), rabbit anti-phospho ERK1/2 (9101, Cell Signaling, USA), rabbit anti-total ERK1/2 (9102, Cell Signaling, USA), rabbit anti-phospho ERK5 (3371, Cell Signaling, USA), rabbit anti-total ERK5 (3372, Cell Signaling, USA), rabbit anti-RUNX2 (sc10758, Santa Cruz Biotech, USA), rabbit anti-Integrin β1 (sc8978, Santa Cruz Biotech, USA), rabbit anti-NFκB (sc298, Santa Cruz Biotech, USA), rabbit anti-PPARγ (sc7196, Santa Cruz Biotech, USA), rabbit anti-GLUT4 (sc7938, Santa Cruz Biotech, USA), rabbit anti-insulin Rβ (sc711, Santa Cruz Biotech, USA) and mouse anti-β-actin (926-42212, Licor, USA). Secondary antibodies included either goat anti-rabbit (800nm) or goat anti-mouse (680nm), which were supplied with the Licor western blotting kit. Proteins were normalized against β-actin to control for differences in protein loading between treatment groups. Protein levels were then normalized against Os-/Veh or Os-/MSDK and compared between groups.

### Measurement of secreted OPG and RANKL

Concentrations of secreted proteins (sOPG and sRANKL) in culture media were measured using human ELISA kits for Osteoprotegerin (Abcam, USA) and Total sRANKL (Enzo Life Science, USA), respectively, per manufacturer's instructions. Culture media was collected immediately before preparing the cell lysates from the bottom chamber of transwell plate (contains hMSCs) or from the layered or monocultures and stored at −20°C until use. Mean concentration changes of sOPG and sRANKL (in pg/mL) and the ratios of sOPG: sRANKL were calculated, normalized against Os-/MSDK and compared between groups.

### Isolation of adipose-derived stem cells in culture and osteogenic differentiation

Mesenchymal stem cells were isolated from the perivascular fraction of adipose tissues obtained from consenting healthy donors and with approval from the Mayo Clinic IRB as described previously [92, 93]. Briefly, fat tissue was digested with 0.075% collagenase Type I (Worthington Biochemicals, USA) for 1.5 h at 37°C and adipocytes were removed from the stromal vascular fraction by low speed centrifugation (400xg for 5 min). Supernatant was aspirated and the cell pellet washed with PBS followed by sieving through 70 and 40 μm cell strainers (BD Biosciences, USA). Cell suspensions (1.0–2.5 ×10^3^ cells/cm^2^) were incubated at 37°C and 5% CO_2_ in Advanced MEM with 5% platelet lysate, PLTMax (MillCreek LifeSciences), 2 U/ml heparin (Novaplus), 2 mM L-glutamine (Invitrogen) and antibiotics (100 U/ml penicillin, 100 g/ml streptomycin, Sigma). Adipose-derived MSCs (AMSCs, passage 7) were seeded at a density of 5000 cells per cm^2^ on 6-well plates and were allowed to adhere overnight and media was then changed to the treatments as described previously; except that some MSCs were incubated in the presence of GSK126, a small molecule inhibitor of the lysine methyltransferase EZH2 and some MSCs were maintained for up to a maximum of 14 days with a media change done every 3-4 days. Alkaline phosphatase activity was assayed at day 7 post-treatment with Os- or Os+ in the absence (vehicle) or presence of MSDK using para-nitrophenyl phosphate (p-NPP) substrate-based chromogenic assay per manufacturer instructions and expressed as ALP activity per unit DNA. DNA was quantified by Hoechst (Sigma) dye measuring the fluorescence at 340/460 nm. Mineralization was analyzed via alizarin red staining at days 8 or 14 as already described and quantified by densitometry using Quantity One software.

### Statistical analysis

A sample size of minimum 10 per group required to detect a significant change in lumber bone density with 80% power was determined by mixed model analysis and based on the means and standard deviations obtained for the serum bone marker data from MOPS [11]. Comparisons of the baseline characteristics between MSDK and placebo groups were performed using Student's two-tailed *t*-test for independent samples with Welch's correction for unequal variances (continuous data) and Fishers exact test (categorical data). Mean changes and percentage mean changes from baseline to month 12 in continuous variables were compared between treatment groups using Student's one-tailed *t*-test for BMD measurements and Student's two-tailed *t*-test for all other endpoints with Welch's correction. Longitudinal analyses were carried out for the continuous variables with repeated measures e.g. serum bone markers, vitamin D_3_ and CRP levels, questionnaires and BP. Generalized linear mixed model (GLMM) approach was used to study the groups, the times, and the interaction between groups and times. In this analysis, groups and times were considered as fixed effects while subjects nested within the treatment groups were considered random. Comparisons of the groups over time were studied using orthogonal contrast. Pearson correlation was performed to analyze the correlation between melatonin, vitamin D_3_ and CRP levels with the bone density, bone markers and morphometric changes. Dairy comments were analyzed using two-tailed Fishers exact test for two categorical outcomes. All statistical testing was carried out using JMP versions 12 (SAS Institute Inc., Cary, NC, USA) and GraphPad Prism version 6 (GraphPad Software, San Diego, CA, USA) for Macintosh. Primary and secondary endpoints were analyzed following the intention-to-treat (ITT) principle. Results were presented as mean ± S.E.M. with significance considered at *p* < 0.05. For *in vitro* assays, all data were normalized against either Os-/Veh or Os-/MSDK and analyzed by one-way ANOVA followed by Bonferroni's post-hoc multiple comparison *t*-test with significance defined as *p* <0.05.
